# Oscillatory neurocomputing with ring attractors: a network architecture for mapping locations in space onto patterns of neural synchrony

**DOI:** 10.1098/rstb.2012.0526

**Published:** 2014-02-05

**Authors:** Hugh T. Blair, Allan Wu, Jason Cong

**Affiliations:** 1 Psychology Department, UCLA, Los Angeles, CA 90095, USA; 2 Computer Science Department, UCLA, Los Angeles, CA 90095, USA

**Keywords:** memory, hippocampus, entorhinal cortex, place cells, grid cells, theta rhythm

## Abstract

Theories of neural coding seek to explain how states of the world are mapped onto states of the brain. Here, we compare how an animal's location in space can be encoded by two different kinds of brain states: *population vectors* stored by patterns of neural firing rates, versus *synchronization vectors* stored by patterns of synchrony among neural oscillators. It has previously been shown that a population code stored by spatially tuned ‘grid cells’ can exhibit desirable properties such as high storage capacity and strong fault tolerance; here it is shown that similar properties are attainable with a synchronization code stored by rhythmically bursting ‘theta cells’ that lack spatial tuning. Simulations of a ring attractor network composed from theta cells suggest how a synchronization code might be implemented using fewer neurons and synapses than a population code with similar storage capacity. It is conjectured that reciprocal connections between grid and theta cells might control phase noise to correct two kinds of errors that can arise in the code: path integration and teleportation errors. Based upon these analyses, it is proposed that a primary function of spatially tuned neurons might be to couple the phases of neural oscillators in a manner that allows them to encode spatial locations as patterns of neural synchrony.

## 1. Introduction

A fundamental aim of computational neuroscience research is to explain how patterns of brain activity store and process information about the world. Here, we consider how the brain encodes information about an animal's location in its environment, by comparing how locations in space can be represented by two kinds of neural activity patterns: *population vectors*, which are patterns of neural firing rates [1–6], versus *synchronization vectors*, which are patterns of phase alignment (or synchrony) among neural oscillators [7–20].

The rodent brain contains several populations of spatially tuned neurons that fire selectively when the animal visits preferred locations in its environment: *place cells* fire at one or a few preferred locations [21], *grid cells* fire at multiple locations forming a hexagonal lattice [22] and *border cells* fire in fixed relationships with environmental boundaries [23–25]. Spatially tuned neurons have been proposed to store population vectors that encode locations in the animal's environment as firing rate patterns [2–6]. However, in rodents, many spatially tuned neurons exhibit rhythmic modulation of their spike trains by 4–12 Hz theta oscillations, which can shift phase against locally recorded electroencephalography rhythms as an animal travels through a cell's firing field [9,26–29]. Such observations have fuelled speculation that theta oscillations might support a temporal code for space [29–34], perhaps by storing synchronization vectors that encode locations in the environment as patterns of neural synchrony [10–20].

Population vector codes for space have been investigated by a class of theoretical models known as ‘continuous attractor networks’ [35–44], in which spatially tuned neurons (such as place or grid cells) are interconnected with one other, so that a stable ‘activity bump’ forms in the network. The activity bump shifts through the network as the animal moves through space, so that the position of the bump in the network reflects the position of the animal in its environment [4,35–44]. Synchronization vector codes have been investigated by a class of theoretical models known as ‘oscillatory interference networks’ [10–20], in which spatially tuned neurons detect position-dependent synchrony among neural oscillators that systematically shift phase against one another as an animal moves through its environment [10–20]. Continuous attractor and oscillatory interference networks are not mutually exclusive categories, because it is possible to implement an oscillatory interference model as a continuous attractor network [17,19,44].

Here, it is shown how a continuous attractor network—namely bank of ring attractors that stores circulating activity bumps—can be alternatively configured to implement either a population vector code stored by spatially tuned grid cells, or a synchronization vector code stored by rhythmically bursting theta cells (which lack spatial tuning). The network exhibits high coding capacity [5,6] and strong fault tolerance [45] under both configurations, but there are a number of fundamental differences between the two coding schemes: the spatial tuning properties and temporal dynamics of neurons in the ring attractors, the reference frame in which activity bumps are measured, and the topology of the mapping from spatial locations onto firing rate space. It is conjectured here that because of these differences, a spatial code might be stored more efficiently by synchronization than population vectors.

## 2. Population versus synchronization coding

A neural code can be specified by a mathematical function, *f* : *D* → *R*, that maps a domain of world states, *D*, onto a range of brain states, *R*. Here, we consider coding functions that can represent an animal's location in space, so *D* will be some finite set of locations in the world that the animal can occupy, and *R* will be some set of brain states that can represent locations in *D*. The mapping from world states onto brain states may be thus written in the form2.1
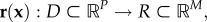
where **x** = [*x*_1_, *x*_2_, *…*, *x_P_*] is a point in a Euclidian space of *P* dimensions (representing a position in the animal's environment), and **r** = [*r*_1_, *r*_2_, *…*, *r_M_*] is a firing rate vector stored by a population of *M* neurons. We shall treat neural populations that are organized to form a network consisting of *N* circular layers, or rings, each containing *M*/*N* neurons. Every ring stores an activity bump that rigidly maintains its shape as it shifts around to occupy different angular positions, or phases, within the ring. States of the network are thus confined to lie upon a manifold that forms an *N*-torus in firing rate space, 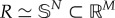. Equation (2.1) may therefore be rewritten as2.2
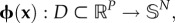
where **φ** = [*ϕ*_1_, *ϕ*_2_, *…*, *ϕ*_*N*_] is a phase vector that lists angular positions for activity bumps in each of the rings. A coding function that implements a mapping of the form in equation (2.2) may be called a *phase code*, because it maps spatial locations 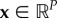 onto phase vectors 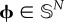.

### (a) Classical population code

Sreenivasan & Fiete [45] have introduced the acronym CPC (classical population code) to label a class of coding functions that map each dimension of a spatial environment onto a single bump phase, so that *N* = *P* in equation (2.2). In the simplest case, we may set *N* = *P* = 1, so equation (2.2) becomes2.3
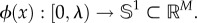


**Figure 1.**
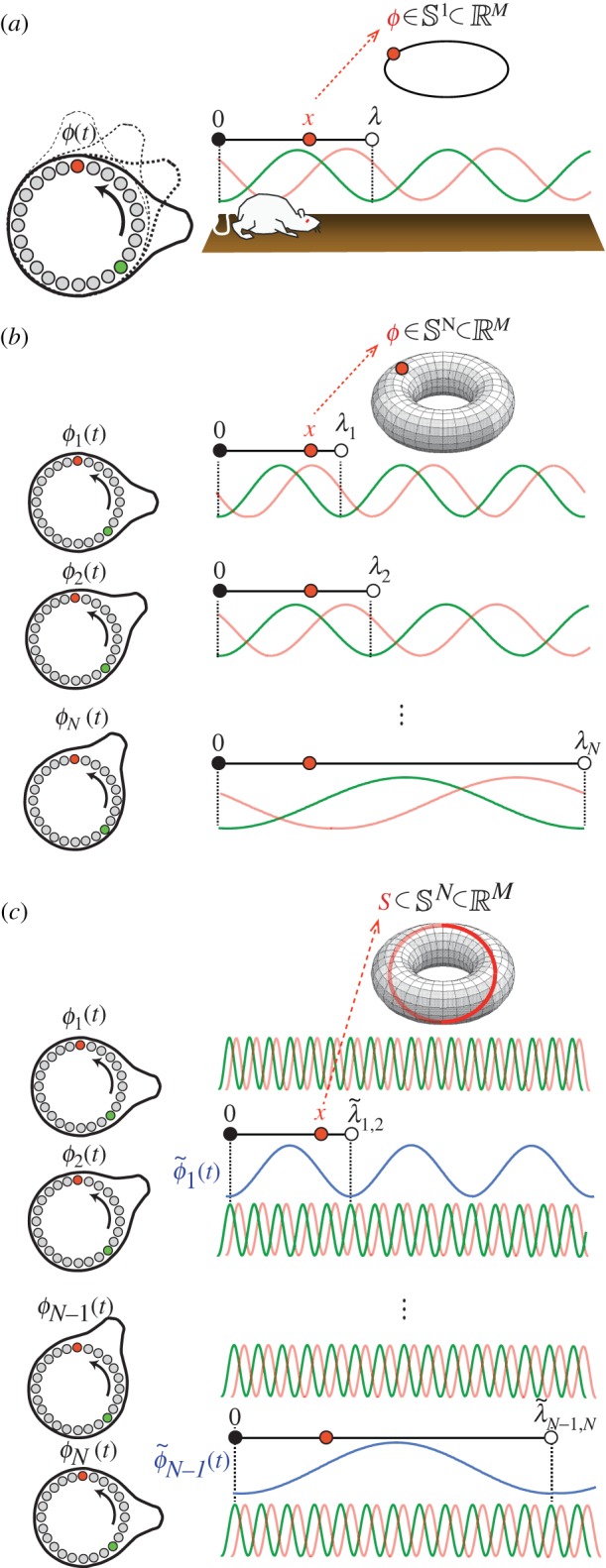
Population versus synchronization coding of positions on a linear track. (*a*) CPC ring containing *M* grid cells (left) with different spatial phases stores an activity bump that maps each position *x* on a linear track to a point *ϕ* lying on a circular manifold in firing rate space. (*b*) GPC network composed from *N* grid cell rings (left) maps each *x* to a point *ϕ* lying on a toroidal manifold in firing rate space. (*c*) OSC network composed from *N* theta cell rings (left) maps each *x* to a circular manifold *S* in firing rate space. (Online version in colour.)

This is a mapping from positions in an interval of length *λ* on a linear track onto a phase angle stored by the angular position of an activity bump in a single ring attractor composed from *M* neurons (figure 1*a*). As the animal moves across the track, the activity bump circulates around the ring at a rate proportional to movement speed. The rate of bump circulation at time *t* is given by2.4
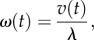
where *ω* is the angular frequency of bump circulation in radians s^−1^, and *v* is the animal's movement speed. Integrating equation (2.4) with respect to time yields an expression for the angular position (or phase) of the activity bump as a function of the animal's position on the track,2.5
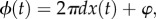
where *d* = 1/*λ* is the spatial frequency, and *φ* is a *spatial reference phase* that denotes the bump's position in the ring when the animal is at position *x* = 0. The animal's position on the track can be decoded from the bump phase by solving for *x,*2.6
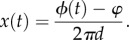
Equation (2.6) measures the position of the activity bump by comparing it against *φ*, a static reference point within the ring attractor. Note that any phase noise in *ϕ*(*t*) would lead to inaccuracies in the decoded position signal, commonly referred to as *path integration errors*.

The activity bump shifts one full cycle (2*π* radians) against *φ* each time the animal traverses a distance *λ* along the track. Hence, neurons in the ring can behave like grid cells, firing periodically at regular intervals along the track (figure 1*a*). However, the CPC network suffers from limitations on storage capacity, because positions on the track are only represented by firing rate vectors that lie upon a circular manifold in firing rate space (figure 1*a*). Because the vast majority of firing rate vectors do not lie upon this circle (and thus do not encode any position on the track), most of the CPC network's potential storage capacity is unused. This is analogous to having a digital computer with many gigabytes of memory capacity, but only being permitted to use a few bytes for data storage. One strategy for expanding the coding capacity is to implement more complex patterns of connectivity among neurons in the ring, thereby allowing more of the representation space to be used [46–48]. An alternative strategy is to divide up the CPC ring to implement a different type of population code.

### (b) Grid population code

Sreenivasan & Fiete [45] have introduced the acronym GPC (grid population code) to label a class of phase codes that possess more degrees of freedom than the spatial environment they represent, so that *N* > *P* in equation (2.2). A GPC network for encoding positions on a linear track may be created by splitting up the single ring in the CPC network described above, dividing its *M* neurons to form a bank of *N* rings, each containing *M*/*N* neurons (figure 1*b*).

In the rodent brain, grid cells are topographically organized into distinct anatomical modules, so that cells residing in the same module have firing fields with the same vertex spacing and orientation (but different translational phases), whereas cells residing in different modules exhibit different spacings and orientations [22,49,50]. If each ring in the GPC network is assigned its own length constant, *λ*_*n*_, then different rings can encode positions on the track at different spatial resolutions (figure 1*b*), and thereby simulate distinct modules composed from grid cells with different vertex spacings. Each ring implements a mapping from track positions to bump positions (equation (2.3)). All rings together implement a mapping of the form2.7
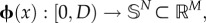
which sends positions on an interval of the track, *x∈*[0, *D*), onto a vector of bump phases, 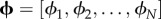, that lie upon an *N*-torus in firing rate space, 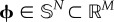 (figure 1*b*). The track segment's length, *D*, depends upon how length constants, **λ** = [*λ*_1_, *λ*_2_, *…*, *λ*_*N*_], are assigned for each ring. Prior work has shown that **λ** can be chosen so that 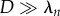 for all *n*; that is, the track interval encoded by the entire GPC network can be far larger than the track interval encoded by any individual ring [5,6].

The multi-ring GPC network maps the animal's position into the same firing rate space as the single-ring CPC network (because the number of neurons has not changed), but now, more of the available coding capacity can be used, because 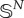 fills up more of the firing rate space than 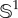. However, this expanded coding capacity comes at a cost, because phase noise can cause more severe consequences under GPC than CPC. Under CPC, phase noise gives rise to path integration errors, which may be defined as inaccuracies in the estimate of an animal's position within its environment. Under GPC, phase noise can give rise to *teleportation errors*, which may be defined as inaccuracies in the estimate of which environment the animal is in.

To see how teleportation errors arise, it may be observed that equation (2.7) maps a low dimensional space of world states (positions on the one-dimensional track) into a high dimensional space of brain states (phase vectors on the *N*-torus). Because dimensionality increases under this transformation, every continuous trajectory on the line, *X⊂*[0, *D*) is guaranteed to map onto a continuous trajectory in phase space, 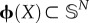. But to decode the rat's position, the mapping of equation (2.7) must be inverted to obtain a mapping from a high to a low dimensional space:2.8
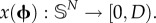
Because dimensionality decreases under this transformation, only a small subset of continuous trajectories in phase space, 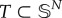, can map back to continuous trajectories on the track, *x*(*T*)⊂[0, *D*). In general, whenever *N* > *P* in equation (2.2), most trajectories in phase space are ‘non-invertible’ in that they map back to non-continuous trajectories through the environment. Consequently, if noise perturbs the GPC network's activity bumps by a small amount that shifts **φ** along a non-invertible trajectory, then this small error in the phase code can generate huge discontinuous ‘jumps’ in the decoded position signal (such as travelling from New York to Paris in a single instant). This is a teleportation error.

Sreenivasan & Fiete [45] have suggested how such errors might be beneficial rather than detrimental. Teleportation magnifies small phase errors in a way that makes them easy to detect, and thus also possibly easier to correct. According to this logic, GPC networks might be highly tolerant to phase noise if they can exploit error correction mechanisms that detect large discontinuities in **x** to correct small errors in **φ**. However, an important constraint upon this strategy is that the cost of the error correction mechanism should not exceed the benefits that it yields in the form of fault tolerance [45]. Hence, error correction networks should be implementable at a low enough cost to justify their yield in performance gains. It is proposed below that one approach to minimizing the cost of error correction might be to use a synchronization code rather than a population code to store phase vectors.

### (c) Oscillatory synchronization code

We shall use the acronym OSC (oscillatory synchronization code) to label a class of phase codes that share many key features of GPC, but store information as synchronization rather than as population vectors. Under the population codes described above (CPC and GPC), the animal's position is decoded by measuring each bump's phase with respect to a fixed reference point, *φ*, as stated by equation (2.6). Each ring performs path integration by shifting its bump against *φ* at an angular frequency that depends linearly upon the animal's running speed, as in equation (2.4). In a GPC network consisting of multiple rings, the slope of this linear relationship can differ among rings (by assigning each ring its own length constant, *λ*_*n*_), but the *y*-intercept must be zero in all rings to maintain a fixed relationship between track positions and bump phases. That is, each bump must stop moving through its ring (*ω* = 0) whenever the animal stops moving across the track (*v* = 0).

To convert the ring attractor network from GPC to OSC, we relax the requirement that all *y*-intercepts must equal zero, but retain the requirement that they must be equal to one another. This change is implemented by assigning the angular frequencies of activity bumps to vary with movement velocity in the manner prescribed by oscillatory interference models [10,11]:2.9
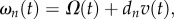
where *Ω* is referred to as the *base frequency* because it denotes the bump's angular frequency when the animal's movement velocity is zero. Equation (2.9) may integrated in time to obtain an expression for the phase of the activity bump in ring *n* as a function of both *x* and *t*:2.10
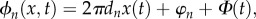
where *Φ*(*t*) is the time integral of the base frequency,2.11
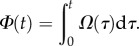


This value shall be referred to as the *temporal reference phase*, because it is the sole component of *ϕ*_*n*_ that varies with *t* independently from *x*. Equations (2.10) and (2.11) assume (without loss of generality) that *Φ* and *x* are both initially zero: *Φ*(0) = *x*(0) = 0.

The presence of *Φ*(*t*) on the right-hand side of equation (2.10) implies that *ϕ*_*n*_ continues to change even when *x* is held fixed; that is, activity bumps continue moving through their rings even when the animal is sitting still. Consequently, neurons in the rings no longer exhibit spatial tuning functions resembling grid cells. Instead, the neurons burst rhythmically as activity bumps circulate around the rings. If *Ω* is assigned a value that lies within the theta frequency band of 4–12 Hz, as in prior oscillatory interference models [10–20], then neurons in the rings burst at a frequency in the theta range. The burst frequency shifts linearly with *v*, at a slope determined by *d_n_*. If *d_n_* differs among rings, then neurons within the same ring always burst at the same frequency, whereas neurons in different rings burst at different frequencies for *v ≠* 0 and at the same frequency for *v* = 0 (because *Φ*(*t*) is equal across all rings). The rodent brain contains ‘theta cells’ that burst rhythmically at velocity-dependent frequencies between 4 and 12 Hz, but lack strong spatial tuning of their firing rates [17,51,52]. In the OSC network, neurons in the ring attractors behave similarly to such theta cells [12,17,19].

If rings are composed from theta cells that do not exhibit spatial tuning, then the animal's position on the track cannot be decoded by measuring bump positions with respect to *φ*, as in equation (2.6). However, track positions can be decoded if the bump positions are measured in a new reference frame. Position may be decoded from phase by solving equation (2.10) for *x* to obtain2.12
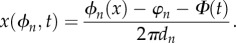


Equation (2.12) measures the position of the activity bump by comparing it not only against *φ*_*n*_ (a static reference in ring *n*), but also against *Φ*(*t*) (a non-stationary reference shared by all rings). It is this non-stationary reference frame for measuring the bump position that distinguishes a synchronization code (such as OSC) from a population code (such as CPC or GPC).

### (d) Decoding position from synchrony

Equation (2.12) implies that to decode positions from phase, *ϕ*_*n*_ must be compared against *Φ*(*t*). To facilitate this, some interference models incorporate a ‘reference oscillator’ assigned a zero length constant (*λ*_0_ = 0), so that its phase is identical to the temporal reference, *ϕ*_0_ = *Φ* [11–16]. Implementing a reference oscillator allows *Φ* to be explicitly observed and measured, but also consumes resources (for example, an additional ring attractor to serve as the reference). An alternative approach is to treat *Φ* as a ‘hidden state’ of the network, which cannot explicitly be measured and thus remains unknown to any external observer or decoder. As long as it is known that rings obey equation (2.9), then the actual value of *Φ* can remain unknown, because position can be decoded by comparing the phase of one bump against another, rather than against *Φ* (strategies for enforcing obedience to equation (2.9) without a reference oscillator will be discussed below). The bump positions in two rings, *i* and *j*, may be compared by subtracting their phases,2.13
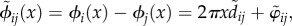
where 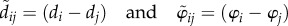. Solving for *x* gives2.14
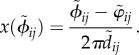
This expression has the same form as equation (2.6), except that instead of comparing the position of a single bump against its reference phase, equation (2.14) decodes the animal's position on the track by comparing the phase alignment between two bumps, 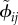, against a reference alignment, 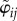 (the alignment between bumps *i* and *j* at *x* = 0). The phase alignment between *i* and *j* shifts one full cycle (2*π* radians) each time the animal traverses a distance 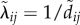 along the track (figure 1*c*).

In an OSC network consisting of *N* ring attractors, there are *N−*1 unique pairs of rings that can be compared with one another; the total number of pairs is (*N*^2^*−N*)/2, but only *N−*1 pairs are non-redundant. Hence, the network represents the animals position as a vector of differences between bump phases, 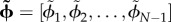. Such a vector of bump phase differences shall henceforth be referred to as a *synchronization vector*, denoted by placing a tilde over the vector of raw bump phases from which it is computed. There are many different ways to define 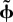, depending upon how bump phases are paired up with one another for comparison. For example, one might arbitrarily assign some specific bump phase, *ϕ*_*i*_, to be a ‘pseudo-reference’ so that 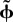 can then be defined as the vector of all phase differences *ϕ*_*i*_ − *ϕ*_*j*_ for which *i* ≠ *j*. An alternative approach, which shall be adopted in the analyses below, is to define the synchronization vector as a first-order difference vector of bump phases,2.15
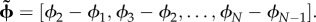


Any 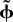 that contains a complete set of non-redundant phase pairings defines a one-to-one mapping from locations in the environment onto the surface of a torus with *N −* 1 dimensions,2.16
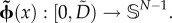


The length, 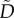, of the track interval encoded by the OSC network depends upon what vector of spatial frequency differences, 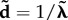, is chosen for the ring attractors, in exactly the same way that value of *D* for the GPC network depends upon what vector of spatial frequencies, **d** = 1/**λ**, is chosen for the ring attractors (see equation (2.7)). So, in principle, any GPC network's spatial frequency vector, **d**_GPC_, could be matched exactly by a corresponding OSC network that contains one additional ring, with spatial frequencies chosen so that 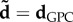. It follows from this that desirable properties of GPC codes, such as their potential for high coding capacity [5,6] and strong fault tolerance [45], can be shared also by OSC codes. However, OSC has one less degree of freedom than GPC (compare equations (2.7) versus (2.16)), and therefore, an OSC network requires one more ring to achieve the same storage capacity as a comparable GPC network. But importantly, ring attractors obey different dynamics in OSC and GPC networks. Simulations presented below indicate that the cost of implementing each ring may be lower for OSC, raising the possibility that OSC may be more efficient than GPC, despite requiring an additional ring.

Unlike population codes, synchronization codes do not implement a one-to-one mapping of spatial locations onto firing rate vectors (equations (2.1) and (2.3)). Instead, the mapping onto firing rate vectors is one-to-many, because the animal's position is represented by the positions of activity bumps with respect to one another (equation (2.14)), rather than with respect to static reference points in their own rings (equation (2.6)). Consequently, the OSC network maps each location in the environment onto a circular manifold *S* in firing rate space (figure 1*c*), containing the set of all rate vectors for which activity bumps are in the same alignment relative to one another, but at different absolute positions in their respective rings. OSC thus possesses a property that GPC lacks: flexibility to represent each location in space by more than one firing rate vector. It shall be conjectured below that this property might make it possible for teleportation errors to be detected and corrected more efficiently under OSC than GPC.

## 3. A ring attractor network for synchronization coding

In this section, we present simulations to show how OSC can be implemented by a network of ring attractors composed from spiking neurons. The ring attractor model described here is adapted from Song & Wang's [53] model of angular path integration by head-direction cells. They described a ring attractor composed from three circular layers—one excitatory and two inhibitory—of integrate-and-fire neurons, with movement of the activity bump controlled by the balance of external driving inputs onto the inhibitory layers [38]; equal drive to both inhibitory layers held the bump still, whereas unequal drive pushed the bump clockwise or anticlockwise (depending upon which layer received the stronger driving input). Here, we modify the original circuit, so that the activity bump circulates in only one direction. The model was implemented using an engine for simulating integrate-and-fire networks in real time on field-programmable gate array (FPGA) microchips [54] (see electronic supplementary material, Methods).

### (a) Ring oscillator circuit

**Figure 2.**
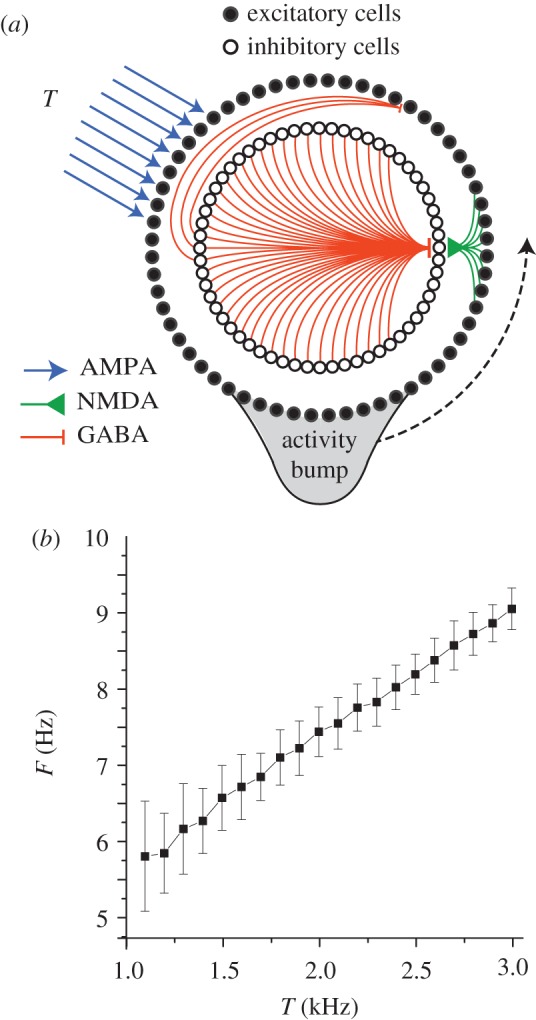
Spiking model of a ring oscillator. (*a*) Two layers of neurons, one excitatory (outer) and the other inhibitory (inner), are interconnected so that an activity bump circulates around the ring at a frequency regulated by Poisson velocity inputs to the excitatory layer. (*b*) Mean ring oscillator frequency in Hz (*y*-axis) versus firing rate, *T*, of Poisson velocity inputs (*x*-axis); error bars show s.d. for average of 100 cycles. (Online version in colour.)

Unidirectional bump circulation requires only one inhibitory layer (rather than two), so in the current model, each ring contains two layers of neurons: one excitatory and the other inhibitory (figure 2*a*). There were 108 integrate-and-fire neurons in each layer, and thus 216 neurons in each ring. Ring layers were interconnected by Gaussian weight vectors (see electronic supplementary material, equation S1). Rates of bump circulation were regulated by a velocity signal represented in the mean firing rates of AMPAergic Poisson spike train inputs onto the excitatory layers of the rings. The *n*th ring's mean Poisson input rate, 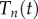, was linearly modulated around a fixed baseline rate, *b*, in proportion to the animal's movement velocity along a preferred vector assigned to that ring. Hence, the *n*th activity bump's circulation frequency is3.1
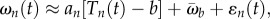
where 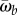 is the mean frequency for *T*(*t*) = *b* (which is identical across rings), *ɛ*_*n*_(*t*) is an error term added to account for frequency noise arising from the Poisson spike inputs, and *a_n_* is the slope of the linear relationship between *ω*(*t*) and *T*(*t*) *− b*. The values of *a_n_* and 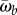 were empirically determined, so that the oscillation frequency varied within the theta band of 6–9 Hz for values of *T* ranging between 1 and 3 kHz (figure 2*b*). The high mean firing rates of the Poisson inputs were intended to represent the summed influence of noisy synaptic inputs from a large number of neurons with velocity-dependent firing rates, as in [53].

### (b) Synchronization coding in two dimensions

If we wish to represent an animal's position in a two-dimensional (open field) environment, then equation (2.16) becomes 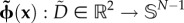, where **x** = (*x*, *y*) is the animal's position within a bounded domain, 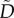, of the environment [11–17]. The scalar spatial frequency, *d_n_* (equations (2.9) and (2.10)), must now be replaced by a Cartesian vector,3.2
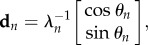
which defines a ‘preferred’ direction, *θ*_*n*_, along which displacement of the animal's position causes the *n*th ring's phase, *ϕ*_*n*_(*t*), to shift against the global reference phase, *Φ*(*t*), with a slope determined by *λ*_*n*_. Equation (2.13) thus becomes3.3
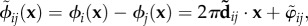
which states that the phase alignment (or synchronization) between two ring oscillators, *i* and *j*, encodes the animal's position along a two-dimensional vector, 
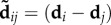
. Simulated ring oscillators were induced to behave this way by assigning the Poisson rate of driving inputs to each ring as *T_n_*(*t*) ≈ *b* + **d***_n_ ·* **v**(*t*) (equation (3.1)), where **v**(*t*) is the animal's movement velocity at time *t*.

**Figure 3.**
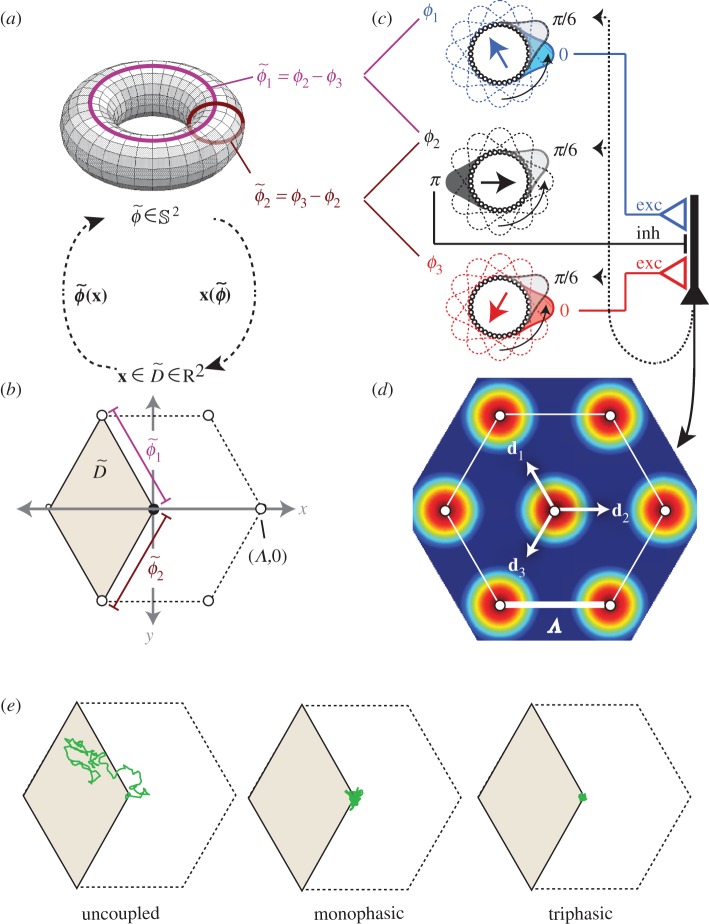
Encoding and decoding of two-dimensional positions by a bank of three ring oscillators. (*a*) The synchronization vector 
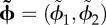
 defines a point on the torus. (*b*) The decoded position lies within a rhomboidal ‘root tile’ (with sides of length *Λ*) that tessellates the plane. (*c*) A grid cell (right) is reciprocally connected with three ring oscillators with spatial frequency vectors **d**_1_, **d**_2_, **d**_3_ (note that in this example, the grid cell receives input from the inhibitory layer of ring 2, and the excitatory layers of rings 1 and 3). (*d*) The rhomboidal root tile occupies one third of the grid cell's hexagonal firing field. (*e*) Decoded position signal exhibits Brownian drift for uncoupled ring oscillators, but only small path integration errors when phase noise is controlled by one (monophasic) or three (triphasic) grid cells. (Online version in colour.)

Here, we implement a minimal oscillatory interference model for grid cells in an open field, consisting of three oscillators [14,15]. Three ring oscillators integrate the animal's movement velocity along different vectors of identical length, *|***d**_1_*|* = *|***d**_2_*|* = *|***d**_3_*|* = 1/*λ*, but oriented 120° apart, *θ*_1_ + 120° = *θ*_2_ = *θ*_3_−120° (figure 3). Substituting these values into equation (3.3) yields a coding function, 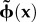, that can be inverted to decode position from synchrony as follows:3.4
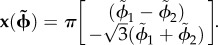

This function decodes a two-dimensional synchronization vector to specify the animal's position within a rhombus-shaped region of the environment, 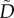, with sides of length *Λ* = 4*π*/√3|**d̃**_*ij*_| (figure 3*c*).

Poisson velocity inputs give rise to independent frequency noise in each ring, denoted by *ɛ*_*n*_ in equation (3.1). This frequency noise is directly analogous with noisy drifting of the activity bumps in continuous attractor models. Integration of this frequency noise produces path integration errors, so that the decoded position signal derived by equation (3.4) deviates from the animal's true position. Because 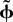 has the same number of dimensions as the environment (two), teleportation errors cannot occur in this minimal grid cell model (strategies for correcting teleportation errors in larger networks are discussed in §3*e*). Figure 3*e* shows how path integration error accumulates in a simulation of three uncoupled ring oscillators. All ring phases were initialized to identical values, *ϕ*_1_(0) = *ϕ*_2_(0) = *ϕ*_3_(0), so the starting position of the simulated animal was at the origin. Identical Poisson inputs ranging from *T* = 1.5 to 2.5 kHz were then delivered constantly to all three rings, encoding a movement velocity of zero, so the ‘true’ position remained fixed at the origin throughout the simulation. Under these conditions, 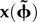 was observed to drift along a Brownian trajectory as error accumulated with time (figure 3*e*, left).

### (c) Phase resetting by grid cells

Because the position estimate is decoded from the synchronization vector, 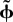, and not from the raw oscillator phases, **φ**, reducing path integration error does not mandate reduction of frequency noise in the rings, but instead merely requires that any frequency noise that is present must be *shared* among the rings, by becoming absorbed into the temporal reference phase, *Φ* [14,55]. This requires some mechanism for coupling the ring oscillators together in a way that allows them to share noise. Here, we propose how grid cells might provide such a mechanism.

Grid cells were simulated by single-compartment neurons that detected synchrony among inputs from all three ring oscillators. Each grid cell received input from the inhibitory layer of one ring, and from and the excitatory layers of the other two rings (figure 3*c*). Input from ring *n* was weighted by a Gaussian vector with a centre phase, *μ*_*n*_, that corresponded to the spatial reference phase, *φ*_*n*_, in equation (2.12) (for explanation, see the electronic supplementary material, equation (S3)). Therefore, assigning the centre phases of the input weights determined which particular alignment of the three activity bumps would produce synchronized input to the grid cell. Specifically, a grid cell's oscillatory inputs from rings *n* = 1, 2, 3 were synchronized when3.5
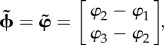
which shall be referred to as the grid cell's *target synchronization vector*. When 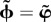, the grid cell was maximally excited to burst along with its synchronized inputs from the three rings. Disregarding path integration error, 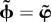 is satisfied at a set of locations which form a hexagonal lattice, and these are the vertices of the grid cell's firing field. The grid cell has a vertex at the origin of the plane if 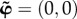, which occurs when 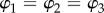. If the spatial reference phase of ring *n* is shifted by an arbitrary offset, *φ*_*n*_ + *Δφ*_*n*_, then the grid field translates in the direction of ring *n*'s frequency vector, *θ*_*n*_, by a distance 
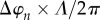
.

Grid cells not only received input from the rings, but also sent feedback projections to the rings, which targeted only the excitatory but not the inhibitory layer. Like feed-forward projections, feedback projections were also weighted by Gaussian vectors, with centre phases tuned so that when a grid cell fired, the system was nudged towards a basin of attraction at the point where 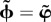 (see electronic supplementary material, figure S1). This recurrent loop between ring oscillators and the grid cell formed a kind of ‘oscillatory attractor’ network, where the grid cell greedily tried to keep itself active by forcing the ring oscillators into the specific alignment that caused it to fire.

To simulate the oscillatory attractor with a single grid cell, the three ring oscillators were initialized with identical bump phases, so that the simulated animal's starting position was initialized at the origin. Centre phases for inputs to the grid cell were assigned so the starting position was also the attractor state, 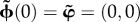. Identical driving inputs of 2 kHz were delivered throughout the simulation to encode a movement velocity of zero (that is, a stationary animal). Simulation showed that the decoded position signal remained well confined to a small neighbourhood surrounding the origin where 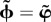 (figure 3*e*, middle), in contrast to the Brownian drift that was observed without coupling. There was still some residual path integration error in the decoded position signal, causing it to fluctuate within a small neighbourhood of the origin. This residual error occurred because resetting of the ring phases by feedback from the grid cell is noisy (it does not reliably reset the phase difference vector to exactly 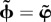 on every cycle), and because phase resetting occurs only once per theta cycle. To reduce within-cycle error accumulation, two additional grid cells were added to the circuit, yielding a total of three grid cells. For the sake of symmetry, each of the three grid cells received its inhibitory input from a different ring oscillator, and received excitatory input from the remaining two rings (see electronic supplementary material, equation (S4)). All three grid cells were assigned to have overlapping grid fields by setting their target synchronization vectors equal to one another, 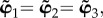 where subscripts index the three grid cells. This caused the three grid cells to fire at identical locations, but they did not fire at identical times; they were assigned to fire on staggered phases of the theta cycle by setting their centre phases so that their spatial reference phase vectors differed by constant offsets: ***φ***_1_ *−* 2*π*3 = ***φ***_2_ = ***φ***_3_ + 2*π*3. Consequently, the three grid cells cooperatively delivered feedback pulses to the ring oscillators on three distinct phases of the theta cycle, an arrangement referred to as *triphasic entrainment.* Under triphasic entrainment, the decoded position signal remained tightly confined near 
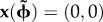
 throughout a 5 s simulation (figure 3*e*, right).

### (d) Microcircuit architecture for a grid module

**Figure 4.**
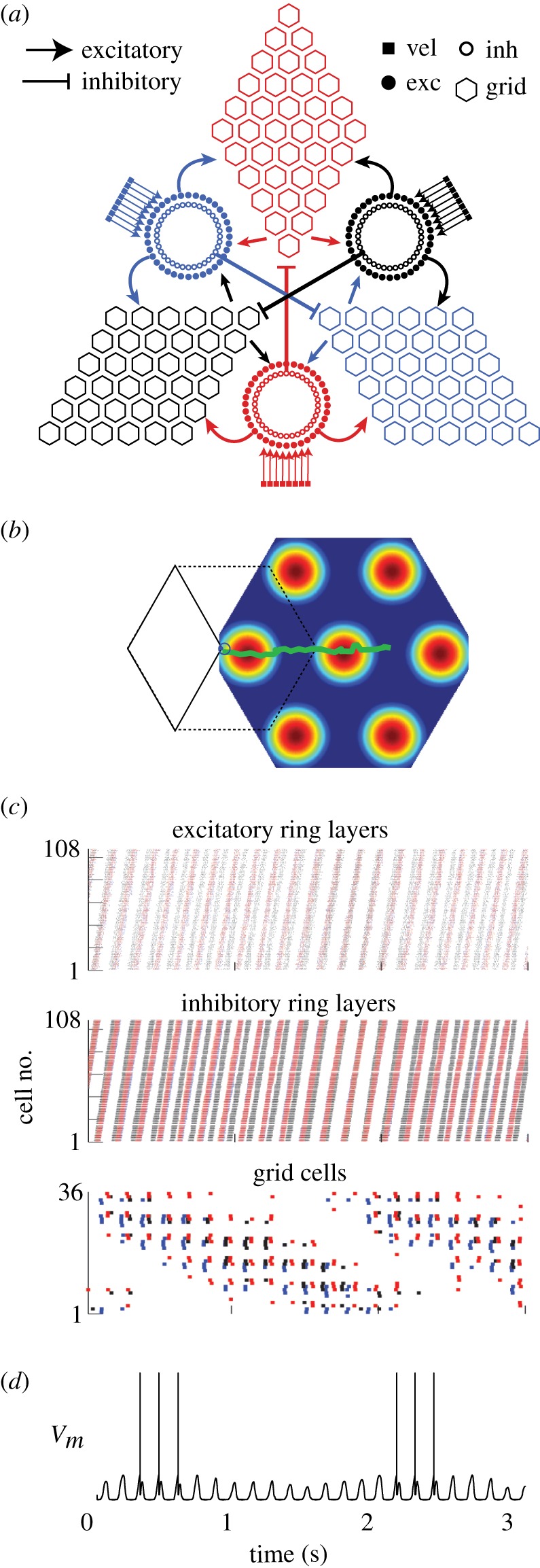
Microcircuit architecture for a grid module. (*a*) Three ring oscillators receive their own independent velocity inputs (vel); the two layers (exc and inh) in each ring are reciprocally interconnected with three 6 × 6 sheets of grid cells. (*b*) Position trajectory decoded from the ring phases during a 3 s simulation in which velocity inputs were assigned to fixed values representing movement along a straight horizontal path. (*c*) Spike rasters for all cells during the simulation shown in (*b*). (*d*) Membrane voltage plotted for a grid cell that fires at vertices shown by the rate map in (*b*). (Online version in colour.)

Figure 3*e* shows how grid cells can hold 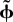 at a fixed attractor point when the animal is stationary. But in a moving animal, 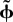 cannot rest at a fixed attractor point because it must follow a trajectory through synchronization space that accurately encodes the animal's navigational trajectory through the environment. This can be achieved by a microcircuit model of a ‘grid module’ consisting of multiple grids cells with different target synchronization vectors (figure 4*a*), so that different grid cells compete with one another to hold the ring oscillators in their own preferred phase alignments. All grid cells in the module have identical vertex spacings and orientations, because they are reciprocally connected to the same triad of ring oscillators. Each module contains three rhomboidal sheets of grid cells. Spatial phases of grid cells within each sheet are evenly distributed over the rhomboidal domain, 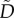 (figure 3*b*). Grid cells at the same row and column position in different sheets have the same spatial phase, but fire on staggered temporal phases of the theta cycle (consistent with recent data [56] showing that simultaneously recorded pairs of entorhinal neurons tend to fire at characteristic phase offsets from one another). Feed-forward weight vectors obeyed the convention that each grid cell received inhibitory input from one ring, and excitatory input from the other two rings. A formula for assigning centre phases that meet these constraints is given by the electronic supplementary material, equation (S6).

Each grid cell sends feedback to all three rings, to reset their phases in such a way that when any given grid cell fires, the synchronization state is pushed towards its own target synchronization vector, 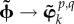 (where *k* indexes the sheet, and *p*, *q* indexes the row and column position of a grid cell within its sheet). This places grid cells at different positions within the same sheet in competition with one another, and at the same position in different sheets in cooperation with one another, to lock the ring phases into their own preferred phase alignments, 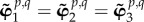. Such competition endows the grid module circuit with heteroclinic stability in synchronization space, so that the system rests ‘uneasily’ in a discrete set of semi-stable synchronization states, each preferred by a different grid cell.

To demonstrate the activity of a single grid module, a 3 s simulation was run in which the Poisson velocity input to the rings was assigned to encode movement in the rightward direction along the horizontal at a constant velocity (figure 4*b*). Neural activity propagated through the rings and the grid sheets as the simulated animal moved along a straight path (figure 4*c*). As in prior oscillatory interference models [10–20], simulated grid cells exhibit hexagonal firing fields, as well as temporal modulation of their spike trains by theta oscillations. Membrane voltages of individual grid cells were qualitatively similar to intracellular recordings of grid cells from behaving rodents [57–59], in that they were modulated by subthreshold theta oscillations when the animal was outside the grid field, and then spikes were generated during passage through grid fields (figure 4*d*). Voltage traces in figure 4*d* do not exhibit the membrane voltage ‘ramps’ that precede spiking in rodent grid cells, but such ramping has been shown to emerge when lateral inhibition is present among grid cells [58], which were not included in the implementation simulated here.

### (e) Correcting teleportation errors among multiple grid modules

If the network contains more than three rings, then neurons can synthesize spatial tuning functions more complex than hexagonal grids via oscillatory interference. For the general case of a post-synaptic cell that fires when its inputs from *N* ring oscillators are synchronized, the firing rate map may be approximated as a two-dimensional spatial envelope function [17,18]:3.6
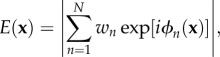
where 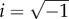, and *w_n_* is the synaptic weight on the input from ring *n*. Equation (3.6) implies that if the weighting coefficients **w** = [*w*_1_, *w*_2_, *…*, *w_N_*], spatial frequencies **D** = [**d**_1_, **d**_2_, *…*, **d***_N_*] and local reference phases are properly chosen, then a neuron that detects synchrony among *N* rings can act as a Fourier synthesizer to generate almost any spatial tuning function, including functions that resemble the firing rate maps of grid, place or border cells [17,18].

If the network is expanded to more than three rings, then 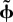 acquires more dimensions than the two-dimensional spatial environment, and teleportation errors can occur. An error correction mechanism must therefore be implemented to prevent teleportation, by constraining the system to follow only invertible phase trajectories. This is tantamount to coupling the rings together so that they share a common temporal reference phase, because invertible trajectories are precisely those for which there exists a function, *Φ*(*t*), that can substituted into equation (2.12) to assure that all ring phases, *ϕ*_*n*_, yield mutually compatible (non-contradictory) descriptions of the decoded movement trajectory along their individual path integration vectors, **d***_n_*. If a solution for *Φ*(*t*) exists given some trajectory *T*, then that trajectory must invertible. Conversely, if *T* is invertible, then a solution for *Φ*(*t*) must exist, but position can be decoded from the bump phases even if the value of *Φ*(*t*) remains hidden from the decoder (equation (3.3)).

These considerations suggest two possible error correction strategies for synchronization coding networks. One approach would be to explicitly represent *Φ*(*t*) by assigning a reference oscillator to encode its value [11–16]; it might then be possible to implement some mechanism that forces a bank of *N* oscillators to follow only trajectories for which *Φ*(*t*) yields a solution to equation (2.12) that gives mutually compatible position estimates across all oscillators. However, we do not know of proven solutions to this reference oscillator coupling problem for arbitrarily large *N*. Another approach would be to ignore the temporal phase, *Φ*(*t*), and focus instead upon the spatial phases of grid cells. If each grid module contains three ring oscillators (as in figure 4*a*), then a network composed from *Z* grid modules (presumably with different orientations and spacings) would contain *N* = 3*Z* ring oscillators. Preventing oscillators from following non-invertible phase trajectories becomes identical to the problem of prohibiting grid cells across modules from encoding trajectories that deviate from one another. A solution to this problem has previously been proposed by Sreenivasan & Fiete [45], who suggested how an error correction network composed from place cells could enforce fault tolerance upon a population vector code stored by grid cells. Their solution involved no reference oscillator, because it was proposed for a population coding network in which *Φ*(*t*) does not exist at all. We see no reason why a similar error correction mechanism could not be implemented in a synchronization coding network, because even though the value of *Φ*(*t*) would not be explicitly represented by such a solution, its existence would be implicitly guaranteed by rigid coupling among grid modules. An interesting question for future study is whether an error correction mechanism similar to that proposed by Sreenivasan & Fiete [45] might be improved upon—that is, made less costly and more efficient—by implementing it under a synchronization code rather than a population code (see §4).

## 4. Discussion

Here, we have shown how a bank of ring attractors can be configured to implement either a population vector code, stored by spatially tuned grid cells, or a synchronization vector code, stored by rhythmically bursting theta cells (which lack spatial tuning). The network exhibits high coding capacity [5,6] and strong fault tolerance [45] under both configurations, but we conjecture that a spatial code might be stored more efficiently by synchronization than population vectors, based on several differences between the two coding schemes: the temporal dynamics of network activity, the reference frame in which activity bumps are measured, and how spatial locations are mapped into firing rate space.

### (a) Potential benefits of synchronization coding

Simulations presented above suggest that because of differing temporal dynamics, a synchronization code (OSC) might be implemented using fewer neurons and synapses than a population code (GPC) with similar storage capacity. This may seem counterintuitive, since OSC requires one more ring than GPC (compare equations (2.7) versus (2.16)). However, under GPC, activity bumps must be able to shift through their rings in either direction (and remain stationary when the animal stops moving), and a standard solution for implementing this functionality in a ring attractor circuit requires three layers of neurons [38,53]. By contrast, under OSC, the bumps only need to shift in one direction, and need never remain stationary. We have shown that this functionality can be implemented by a reduced attractor model with only two layers. Further study is needed to verify whether coding capacity is fully preserved under this modification under comparable noise conditions, but if each ring in the network can be built with fewer neurons and synapses, then it is possible that an OSC network might be implemented at a lower cost of resources than a comparable GPC network, with the cost savings proportional to the number of rings.

As the number of rings in the network increases, so too does the need for an efficient mechanism to prevent teleportation errors by controlling phase noise. Oscillatory interference models have sometimes been criticized as implausible on the grounds of their vulnerability to phase noise, but such criticisms are misplaced, because any high dimensional phase code exhibits ‘pathological sensitivity to noise’ [45], regardless of whether it is stored by population vectors (as in GPC) or synchronization vectors (as in OSC). Prior studies have proposed plausible mechanisms for noise reduction in oscillatory interference models [16,55] as well as continuous attractor models [45]. Here, we proposed how phase errors in ring oscillators might be corrected through entrainment of theta cells by grid cells (figure 3*c*), and how reciprocal connections between grid and theta cells could implement an ‘oscillatory attractor network’ that exhibits heteroclinic stability in phase space (figure 4*a*). Under such an arrangement, teleportation errors might be corrected by rigidly coupling multiple grid modules together, possibly by adapting a prior solution in which place cells enforce fault tolerance upon a population code stored by grid cells [45]. It remains an open question whether alternative error correction schemes might be possible in synchronization coding networks, and what efficiency gains might be realized by such alternatives. Perhaps by exploiting reference oscillators, or by capitalizing upon the ability to represent a single location by multiple firing rate vectors, synchronization coding might offer possibilities for highly efficient error correction. Further research is needed to compare how the performance of different error correction mechanisms scale with their cost.

Population vectors are decoded by measuring the position of each activity bump within the ring that stores it, whereas synchronization vectors are decoded by measuring the position of an activity bump in one ring with respect to the bump in another (which is tantamount to detecting synchrony among the oscillations generated by different rings). When bump circulation is modulated around a well-defined base frequency (such as theta), the code is endowed with a temporal structure that may be beneficial in a number ways; for example, the carrier frequency defines a specific observational time frame for decoders to interpret the position signal (the length of a single theta cycle), and spike trains acquire a temporal structure that may be beneficial for driving spike-timing-dependent neural plasticity processes that are thought to support learning and memory functions in biological networks [30,32,33,59].

### (b) Empirical evidence for synchronization coding

Synchronization coding offers parsimonious explanations for certain temporal properties of spatially tuned neurons in rodents, such as theta rhythmicity and phase precession of spike trains against the local field potential [9–20,26–32,60]. In rats, pharmacological blockade of theta rhythm disrupts the spatial tuning of grid cells [61,62], as would be expected if grid cells derive their spatial tuning from synchronization of theta oscillations. However, place cells continue to exhibit spatial firing after similar disruptions of theta [62,63], raising questions about whether theta is essential for all spatial coding. In mammals other than rodents, such as bats [64], place and grid cells appear not to be strongly modulated by theta oscillations (but see [65]). Such findings raise further questions about whether theta oscillations play an essential role in spatial coding. However, from the standpoint of synchronization coding, there is nothing special about the theta frequency. The base frequency for the synchronization code (*Ω*(*t*) in equation (2.9)) need not lie in the theta band; it could be shifted to a higher or lower frequency without altering the essential properties of the code. *In vitro* studies of bat neurons suggest that these neurons may oscillate at a frequency lower than theta, so it is possible that they might implement a synchronization code in a different frequency band [66].

All synchronization coding models make one essential prediction: the brain should contain oscillators that shift phase against one another as a function of an animal's position in its environment. This prediction is well supported by data from rodents running on linear tracks, where field potential theta oscillations shift phase against the spikes of place cells [9,26,28,29], grid cells [27,67] and interneurons [29,68] as a function of the animal's position on the track. In two-dimensional open field environments, theta cell burst frequencies have been reported to vary with a rat's movement direction in a manner consistent with synchronization coding [17]. But phase precession of place cell spikes against the field potential in open fields seems to depend upon the distance the animal has travelled through a place cell's firing field, regardless of movement direction [26–28]. This is problematic, because if the animal's allocentric position in two-dimensional space is encoded by synchrony among theta oscillators, then phase precession should be sensitive to movement direction. Burgess [14] has proposed a possible explanation for this discrepancy, showing that phase precession would not depend upon direction if place and grid cells detected synchrony among theta oscillators with correlated amplitude and frequency. But if theta oscillations that provide input to these cells were recorded at their source of origin (rather than in a post-synaptic place or grid cell), they would still be predicted to shift phase against one another in a manner that depends upon movement direction, and this has not yet been observed. Further experiments are thus warranted to investigate whether any theta-modulated regions in the rodent brain produce oscillations that shift phase against one another in the manner that could support a synchronization code for space.

## References

[1] Pouget A Dayan P Zemel RS 2003 Inference and computation with population codes. Annu. Rev. Neurosci. 26, 381–410. (10.1146/annurev.neuro.26.041002.131112) 12704222

[2] Wilson MA McNaughton BL 1993 Dynamics of the hippocampal ensemble code for space. Science 261, 1055–1058. (10.1126/science.8351520) 8351520

[3] Zhang K Sejnowski TJ 1999 Neuronal tuning: to sharpen or broaden? Neural Comput. 11, 75–84. (10.1162/089976699300016809) 9950722

[4] McNaughton BL Battaglia FP Jensen O Moser EI Moser MB 2006 Path integration and the neural basis of the ‘cognitive map’. Nat. Rev. Neurosci. 7, 663–678. (10.1038/nrn1932) 16858394

[5] Fiete IR Burak Y Brookings T 2008 What grid cells convey about rat location? J. Neurosci. 28, 6858–6871. (10.1523/JNEUROSCI.5684-07.2008) 18596161 PMC6670990

[6] Mathis A Herz AV Stemmler M 2012 Optimal population codes for space: grid cells outperform place cells. Neural Comput. 24, 2280–2317. (10.1162/NECO_a_00319) 22594833

[7] Miall C 1989 The storage of time intervals using oscillating neurons. Neural Comput. 1, 359–371. (10.1162/neco.1989.1.3.359)

[8] Hopfield JJ Brody CD 2001 What is a moment? Transient synchrony as a collective mechanism for spatiotemporal integration. Proc. Natl Acad. Sci. USA 98, 1282–1287. (10.1073/pnas.98.3.1282) 11158631 PMC14746

[9] O'Keefe J Recce ML 1993 Phase relationship between hippocampal place units and the EEG theta rhythm. Hippocampus 3, 317–330. (10.1002/hipo.450030307) 8353611

[10] Burgess N Barry C Jeffery KJ O'Keefe J 2005 A grid and place cell model of path integration utilizing phase precession versus theta. In First Annual Conf. on Computational Cognitive Neuroscience, Washington, DC. (http://posters.f1000.com/PosterList?posterID=225)

[11] Burgess N Barry C O'Keefe J 2007 An oscillatory interference model of grid cell firing. Hippocampus 17, 801–812. (10.1002/hipo.20327) 17598147 PMC2678278

[12] Hasselmo ME Giocomo LA Zilli EA 2007 Grid cell firing may arise from interference of theta frequency membrane potential oscillations in single neurons. Hippocampus 17, 1252–1271. (10.1002/hipo.20374) 17924530 PMC2408670

[13] Giocomo LM Zilli EA Fransén E Hasselmo ME 2007 Temporal frequency of subthreshold oscillations scales with entorhinal grid cell field spacing. Science 315, 1719–1722. (10.1126/science.1139207) 17379810 PMC2950607

[14] Burgess N 2008 Grid cells and theta as oscillatory interference: theory and predictions. Hippocampus 18, 1157–1174. (10.1002/hipo.20518) 19021256 PMC3196519

[15] Hasselmo ME 2008 Grid cell mechanisms and function: contributions of entorhinal persistent spiking and phase resetting. Hippocampus 18, 1213–1229. (10.1002/hipo.20512) 19021258 PMC2614862

[16] Zilli EA Hasselmo ME 2010 Coupled noisy spiking neurons as velocity-controlled oscillators in a model of grid cell spatial firing. J. Neurosci. 30, 13 850–13 860. (10.1523/JNEUROSCI.0547-10.2010) 20943925 PMC2978507

[17] Welday AW Shlifer IG Bloom ML Zhang K Blair HT 2011 Cosine directional tuning of theta cell burst frequencies: Evidence for spatial coding by oscillatory interference. J. Neurosci. 31, 16 157–16 176. (10.1523/JNEUROSCI.0712-11.2011) PMC3758572 22072668

[18] Monaco JD Knierim JJ Zhang K 2011 Sensory feedback, error correction, and remapping in a multiple oscillator model of place-cell activity. Front. Comput. Neurosci. 5, 39 (10.3389/fncom.2011.00039) 21994494 PMC3182374

[19] Blair HT Gupta K Zhang K 2008 Conversion of a phase- to a rate-coded position signal by a three-stage model of theta cells, grid cells, and place cells. Hippocampus 18, 1239–1255. (10.1002/hipo.20509) 19021259 PMC2814603

[20] Zilli EA 2012 Models of grid cell spatial firing published 2005–2011. Front. Neural Circuits 6, 16 (10.3389/fncir.2012.00016) 22529780 PMC3328924

[21] O'Keefe J Dostrovsky J 1971 The hippocampus as a spatial map. Preliminary evidence from unit activity in the freely-moving rat. Brain Res. 34, 171–175. (10.1016/0006-8993(71)90358-1) 5124915

[22] Hafting T Fyhn M Molden S Moser MB Moser EI 2005 Microstructure of a spatial map in the entorhinal cortex. Nature 436, 801–806. (10.1038/nature03721) 15965463

[23] Solstad T Boccara CN Kropff E Moser MB Moser EI 2008 Representation of geometric borders in the entorhinal cortex. Science 322, 1865–1868. (10.1126/science.1166466) 19095945

[24] Savelli F Yoganarasimha D Knierim JJ 2008 Influence of boundary removal on the spatial representations of the medial entorhinal cortex. Hippocampus 18, 1270–1282. (10.1002/hipo.20511) 19021262 PMC3007674

[25] Lever C Burton S Jeewajee A O'Keefe J Burgess N 2009 Boundary vector cells in the subiculum of the hippocampal formation. J. Neurosci. 29, 9771–9777. (10.1523/JNEUROSCI.1319-09.2009) 19657030 PMC2736390

[26] Skaggs WE McNaughton BL Wilson MA Barnes CA 1996 Theta phase precession in hippocampal neuronal populations and the compression of temporal sequences. Hippocampus 6, 149–172. (10.1002/(SICI)1098-1063(1996)6:2<149::AID-HIPO6>3.0.CO;2-K) 8797016

[27] Hafting T Fyhn M Bonnevie T Moser MB Moser EI 2008 Hippocampus-independent phase precession in entorhinal grid cells. Nature 453, 1248–1252. (10.1038/nature06957) 18480753

[28] Huxter JR Senior TJ Allen K Csicsvari J 2008 Theta phase-specific codes for two-dimensional position, trajectory and heading in the hippocampus. Nat. Neurosci. 11, 587–594. (10.1038/nn.2106) 18425124

[29] Geisler C Robbe D Zugaro M Sirota A Buzsáki G 2007 Hippocampal place cell assemblies are speed-controlled oscillators. Proc. Natl Acad. Sci. USA 104, 8149–8154. (10.1073/pnas.0610121104) 17470808 PMC1876586

[30] Jensen O Lisman JE 2000 Position reconstruction from an ensemble of hippocampal place cells: contribution of theta phase coding. J. Neurophysiol. 83, 2602–2609. 10805660 10.1152/jn.2000.83.5.2602

[31] Huxter J Burgess N O'Keefe J 2003 Independent rate and temporal coding in hippocampal pyramidal cells. Nature 425, 828–832. (10.1038/nature02058) 14574410 PMC2677642

[32] Harris KD Csicsvari J Hirase H Dragoi G Buzsaki G 2003 Organization of cell assemblies in the hippocampus. Nature 424, 552–556. (10.1038/nature01834) 12891358

[33] Buzsáki G 2006 Rhythms of the brain. New York, NY: Oxford University Press.

[34] Düzel E Penny WD Burgess N 2010 Brain oscillations and memory. Curr. Opin. Neurobiol. 20, 143–149. (10.1016/j.conb.2010.01.004) 20181475

[35] Amit DJ 1989 Modeling brain function: the world of attractor neural networks. Cambridge, UK: Cambridge University Press.

[36] McNaughton BL 1996 Deciphering the hippocampal polyglot: the hippocampus as a path integration system. J. Exp. Biol. 199, 173–185. 8576689 10.1242/jeb.199.1.173

[37] Samsonovich A McNaughton BL 1997 Path integration and cognitive mapping in a continuous attractor neural network model. J. Neurosci. 17, 5900–5920. 9221787 10.1523/JNEUROSCI.17-15-05900.1997 PMC6573219

[38] Zhang K 1996 Representation of spatial orientation by the intrinsic dynamics of the head-direction cell ensemble: a theory. J. Neurosci. 16, 2112–2126. 8604055 10.1523/JNEUROSCI.16-06-02112.1996 PMC6578512

[39] Fuhs MC Touretzky DS 2006 A spin glass model of path integration in rat medial entorhinal cortex. J. Neurosci. 26, 4266–4276. (10.1523/JNEUROSCI.4353-05.2006) 16624947 PMC6674007

[40] Guanella A Kiper D Verschure P 2007 A model of grid cells based on a twisted torus topology. Int. J. Neural Syst. 17, 231–240. (10.1142/S0129065707001093) 17696288

[41] Burak Y Fiete IR 2009 Accurate path integration in continuous attractor network models of grid cells. PLoS Comput. Biol. 5, e1000291 (10.1371/journal.pcbi.1000291) 19229307 PMC2632741

[42] Navratilova Z Giocomo LM Fellous J-M Hasselmo ME McNaughton BL 2012 Phase precession and variable spatial scaling in a periodic attractor map model of medial entorhinal grid cells with realistic after-spike dynamics. Hippocampus 22, 772–789. (10.1002/hipo.20939) 21484936

[43] Mhatre H Gorchetchnikov A Grossberg S 2012 Grid cell hexagonal patterns formed by fast self-organized learning within entorhinal cortex. Hippocampus 22, 320–334. (10.1002/hipo.20901) 21136517

[44] Hasselmo ME Brandon MP 2012 A model combining oscillations and attractor dynamics for generation of grid cell firing. Front. Neural Circuits 6, 30 (10.3389/fncir.2012.00030) 22654735 PMC3361022

[45] Sreenivasan S Fiete I 2011 Grid cells generate an analog error-correcting code for singularly precise neural computation. Nat. Neurosci. 14, 1330–1337. (10.1038/nn.2901) 21909090

[46] Roudi Y Treves A 2006 Localized activity profiles and storage capacity of rate-based autoassociative networks. Phys. Rev. E Stat. Nonlin. Soft Matter Phys. 73, 061904 (10.1103/PhysRevE.73.061904) 16906861

[47] Anishchenko A Treves A 2006 Autoassociative memory retrieval and spontaneous activity bumps in small-world networks of integrate-and-fire neurons. J. Physiol. Paris 100, 225–236. (10.1016/j.jphysparis.2007.01.004) 17320359

[48] Scarpetta S Giacco F 2013 Autoassociative memory of phase-coded spatiotemporal patterns in leaky integrate and fire networks. J. Comput. Neurosci. 34, 319–336. (10.1007/s10827-012-0423-7) 23053861 PMC3605499

[49] Barry C Hayman R Burgess N Jeffery KJ 2007 Experience-dependent rescaling of entorhinal grids. Nat. Neurosci. 10, 682–684. (10.1038/nn1905) 17486102

[50] Stensola H Stensola T Solstad T Frøland K Moser MB Moser EI 2012 The entorhinal grid map is discretized. Nature 492, 72–78. (10.1038/nature11649) 23222610

[51] Rivas J Gatztelu JM García-Austt E 1996 Changes in hippocampal cell discharge patterns and theta rhythm spectral properties as a function of walking velocity in the guinea pig. Exp. Brain Res. 108, 113–118. (10.1007/BF00242908) 8721159

[52] Jeewajee A Barry C O'Keefe J Burgess N 2008 Grid cells and theta as oscillatory interference: electrophysiological data from freely moving rats. Hippocampus 18, 1175–1185. (10.1002/hipo.20510) 19021251 PMC3173868

[53] Song P Wang XJ 2005 Angular path integration by moving ‘hill of activity’: a spiking neuron model without recurrent excitation of the head-direction system. J. Neurosci. 25, 1002–1014. (10.1523/JNEUROSCI.4172-04.2005) 15673682 PMC6725619

[54] Blair HT Cong J Wu D 2013 FPGA simulation engine for customized construction of neural microcircuits. In Proc. IEEE/ACM Int. Conf. on Computer-Aided Design (ICCAD), San Jose, USA, 18–21 November 2013. 10.1109/FCCM.2013.22 PMC4288851 25584120

[55] Zilli EA Yoshida M Tahvildari B Giocomo LM Hasselmo ME 2009 Evaluation of the oscillatory interference model of grid cell firing through analysis and measured period variance of some biological oscillators. PLoS Comput. Biol. 5, e1000573 (10.1371/journal.pcbi.1000573) 19936051 PMC2773844

[56] Brandon MP Bogaard AR Schultheiss NW Hasselmo ME 2013 Segregation of cortical head direction cell assemblies on alternating θ cycles. Nat. Neurosci. 16, 739–748. (10.1038/nn.3383) 23603709 PMC3703458

[57] Domnisoru C Kinkhabwala AA Tank DW 2013 Membrane potential dynamics of grid cells. Nature 495, 199–204. (10.1038/nature11973) 23395984 PMC4099005

[58] Schmidt-Heiber C Häusser M 2013 Cellular mechanisms of spatial navigation in the medial entorhinal cortex. Nat. Neurosci. 6, 325–331. (10.1038/nn.3340) 23396102

[59] Hansel D Mato G Meunier C 1995 Synchrony in excitatory neural networks. Neural Comput. 7, 307–337. (10.1162/neco.1995.7.2.307) 8974733

[60] Burgess N O'Keefe J 2011 Models of place and grid cell firing and theta rhythmicity. Curr. Opin. Neurobiol. 21, 734–744. (10.1016/j.conb.2011.07.002) 21820895 PMC3223517

[61] Brandon MP Bogaard AR Libby CP Connerney MA Gupta K Hasselmo ME 2011 Reduction of theta rhythm dissociates grid cell spatial periodicity from directional tuning. Science 332, 595–599. (10.1126/science.1201652) 21527714 PMC3252766

[62] Koenig J Linder AN Leutgeb JK Leutgeb S 2011 The spatial periodicity of grid cells is not sustained during reduced theta oscillations. Science 332, 592–595. (10.1126/science.1201685) 21527713

[63] Mizumori SJ McNaughton BL Barnes CA Fox KB 1989 Preserved spatial coding in hippocampal CA1 pyramidal cells during reversible suppression of CA3c output: evidence for pattern completion in hippocampus. J. Neurosci. 9, 3915–3928. 2585060 10.1523/JNEUROSCI.09-11-03915.1989 PMC6569931

[64] Yartsev MM Witter MP Ulanovsky N 2011 Grid cells without theta oscillations in the entorhinal cortex of bats. Nature 479, 103–107. (10.1038/nature10583) 22051680

[65] Barry C Bush D O'Keefe J Burgess N 2012 Models of grid cells and theta oscillations. Nature 488, E1–E2. (10.1038/nature11276) 22859210

[66] Heys JG MacLeod KM Moss CF Hasselmo ME 2013 Bat and rat neurons differ in theta-frequency resonance despite similar coding of space. Science 340, 363–367. (10.1126/science.1233831) 23599495

[67] Maurer AP Cowen SL Burke SN Barnes CA McNaughton BL 2006 Phase precession in hippocampal interneurons showing strong functional coupling to individual pyramidal cells. J. Neurosci. 26, 13 485–13 492. (10.1523/JNEUROSCI.2882-06.2006) PMC6674718 17192431

[68] Climer JR Newman EL Hasselmo ME 2013 Phase coding by grid cells in unconstrained environments: two-dimensional phase precession. Eur. J. Neurosci. 38, 2526–2541. (10.1111/ejn.12256) 23718553 PMC3912569

